# A review of 151 cases of pediatric noncystic fibrosis bronchiectasis in a tertiary care center

**DOI:** 10.4103/1817-1737.30354

**Published:** 2007

**Authors:** Hanaa Hasan Banjar

**Affiliations:** *Department of Pediatrics, King Faisal Specialist Hospital and Research Centre (KFSH and RC), Saudi Arabia*

**Keywords:** Bronchiectasis, chest infection, developing countries

## Abstract

**OBJECTIVE:**

This study was conducted to review the etiological factors and diseases associated with pediatric noncystic fibrosis bronchiectasis in a tertiary care center in Saudi Arabia.

**MATERIALS AND METHODS:**

A retrospective review of all patients with confirmed noncystic fibrosis (Non-CF) bronchiectasis by chest X-ray and/or CT chest in a pulmonary clinic during the period 1993–2005 at a tertiary care center in Riyadh.

**RESULTS:**

A total of 151 cases were diagnosed as Non-CF bronchiectasis. Seventy-five (49.7%) were male, 76 (50.3%) were female; 148 (98%) are alive and 3 (2%) died. The southwestern regions constituted 72 (50%) of the cases. There was a period of (5 ± 3.2) years between the start of symptoms and diagnosis of bronchiectasis. More than two-thirds of the patients had cough, tachypnea, wheezing, sputum production and failure to thrive. Ninety-one (60%) had associated diseases: Pulmonary diseases in 48 (32%), immunodeficiency in 27 (18%), central nervous system anomalies in 10 (7%), cardiac in 10 (7%) and asthma in 103 (68%) of the patients. Left lower lobe was commonly involved in 114 (76%) patients. Sixty-eight (67%) were found to have sinusitis. More than two-thirds of patients had two or more associated diseases. Forty-nine (32%) developed gastroesophageal reflux. Hemophilus influenza was cultured in 56 (37%), strept pneumoniae in 25 (17%) and pseudomonas aeruginosa in 24 (16%) of the patients. Eighty percent of the patients who had pulmonary function test had abnormal changes. Disease progression was related to development of symptoms before 5 years of age, persistent atelectasis and right lower lobe involvement (*P*< 0.05).

**CONCLUSION:**

Non-CF bronchiectasis should be included in the differential diagnosis of recurrent chest infection in Saudi Arabia. Early diagnosis and identification of associated diseases is needed to prevent progression of the disease.

Bronchiectasis was called an orphan disease for the last two decades as its incidence decreased markedly and became an uncommon clinical entity among adults and children in developed countries.[1] It is defined as a permanent dilatation of the bronchi,[2] which typically involves the second to sixth order of segmental bronchi.[2] It was first described by La¸nnec in 1819 based on examination of postmortem specimens.[3] Bierrring studied 151 patients in Copenhagen, following pneumonia, and found only 1 child (0.7%) to have bronchiectasis.[4] Ruberman and colleagues evaluated 69 patients with persistent abnormalities on chest radiographs by bronchoscopy.[5] Out of 1,711 young adults (18 to 25 years of age) treated for pneumonia at a U.S. army hospital, 29 (1.7%) were found to have bronchiectatic changes.[5] Fileld noted a dramatic decrease in admission rates for bronchiectasis at five British hospitals from an average of 48 per 10,000 in 1952 to 10 per 10,000 total pediatric admissions in 1960.[6] She speculated that improved treatment for lower respiratory tract infections was made possible by the increased availability of broad-spectrum antibiotics during that period.[6] Other contributing factors include the prevention of measles and pertussis through immunization and the marked decrease in primary pulmonary tuberculosis in the pediatric population which was brought about by better public health measures and improved treatment regimens for this disease.[3] The incidence of childhood bronchiectasis has been documented in the literature to have an ongoing decline.[2] Clark summarized many series from 1900-1950s and considered his own report of 116 children in 1963.[7] He noted that half of the children developed bronchiectasis following severe pneumonia and estimated the annual incidence of bronchiectasis to be 1.06 per 10,000 children. Most series indicated a male/female ratio of about 1:1.4.[1–6]

Reports of pediatric bronchiectasis from the Arab world are scarce. Dawson *et al.* from United Arab Emirates[8] reported 45 children with severe lung disease; bronchiectasis was a major cause, followed by cystic fibrosis and congenital lung disorder. The indigenous population, particularly, was found to be at risk to develop such disease.[8] Studies from Saudi Arabia on bronchiectasis were heterogeneous. Bronchiectasis post-tracheoesophageal fistula repair was reported in 7 cases, mainly due to gastric and colonic replacement and associated congenital anomalies.[9] In another study, bronchiectasis due to lipid aspiration was reported in 6 children that was resistant to medical treatment but improved after surgical resection.[10] Three out of 28 patients with late presentation of foreign body aspiration developed bronchiectasis.[11] In one study for adults with chronic cough,[12] 5% of 81 patients were found to have bronchiectasis, 26% had asthma, 60% had rhinosinusitis, 9% had gastroesophageal reflux disease and 8% had post-infection cough. The impression was that extra-pulmonary causes such as rhinosinusitis were missed, which is considered benign and rarely requires specialized investigations and is easily treated.

In this report, we present the experience of a tertiary care center in Saudi Arabia on childhood bronchiectasis and review the etiological factors and diseases associated with noncystic fibrosis bronchiectasis.

## Materials and Methods

A retrospective review of charts was done for all patients referred to the pulmonary clinic for evaluation of recurrent chest infection during the period January 1993 to August 2005 at King Faisal Specialist Hospital and Research Center (KFSH and RC) in Riyadh region, which is considered a tertiary care center for referral of complicated cases in Saudi Arabia. Only patients with confirmed bronchiectasis on chest X-rays and/or computerized tomography (CT) scan of the chest (CT) were included in the study. The objective of this study was to present the demographic radiological patterns, associated diseases and pulmonary function test (PFT) data that are associated with this disease.

### Patient investigations

All patients with confirmed bronchiectasis had the following tests done: Respiratory cultures from sputum or from nasopharyngeal aspirates if unable to produce sputum for culture and sensitivity; PPD skin test; sputum for acid fast bacilli (AFB) stain and AFB culture, or gastric aspirates instead of sputum for children who are unable to produce sputum; barium swallow to rule out vascular ring or tracheoesophageal fistula (TEF); sinus CT for those who presented with persistent rhinorrhea for more than 3 months.

For patients who had family history of bronchiectasis, nasal brush by ear, nose and throat specialist (ENT) or biopsy of airway endothelium for electron microscopy to rule out immotile cilia syndrome was done. (Only structural abnormalities were examined.) Pulmonary function test was done for patients >5 years of age and able to comprehend to test maneuvers. Associated diseases were investigated according to presenting symptoms or type of referrals. (For example, magnetic resonance or CT brain was done in patients with cerebral palsy or central nervous system abnormalities.)

### Statistical analysis

SPSS program for Windows (release 11.0.0) was used for data analysis. Chi-square (χ^2^) was used to compare categorical variables. Results were presented at a level of significance of *P* ≤ 0.05

### Definitions

*Progression of disease* is a qualitative measurement defined as radiological deterioration with more lobes involved in addition to clinical deterioration with increased sputum production, cough and/or fever.

*PFT severity* is a quantitative measurement of airflow in PFT. *Mild lung changes* are defined as forced expiratory volume in one second (FEV1) = 65-75% of predicted values.

*Moderate lung changes* are defined as FEV1 = 55-65% of predicted values.

*Severe lung changes* are defined as FEV1 <55% of predicted values.

### Patient management

All confirmed cases of bronchiectasis were screened for cystic fibrosis by sweat chloride test, PPD skin test, respiratory cultures for virology, acid-fast bacilli and other pathogenic bacteria. They were followed every 1-3 month(s) according to the severity of their disease. They were taught how to do regular chest physiotherapy to mobilize secretion and how to take Ventolin and inhaled steroid according to their need. Antibiotic treatment orally or intravenously is advised during exacerbation of their symptoms (as increase in cough or sputum production, change in the color of their sputum to yellowish or greenish or respiratory distress).

Patients who showed clinical deterioration in the form of recurrent fever or increase in sputum or cough or radiological deterioration in the form of involvement of another lobe with bronchiectatic changes or PFT deterioration in all parameters were admitted to the hospital for intensive chest physiotherapy, airway clearance and I.V. antibiotics according to bacterial organism from the respiratory cultures for approximately 7-10 days, in addition to inhalation treatment with albuterol and inhaled steroid.

Lobectomy was done when medical treatment failed to stabilize PFT and radiological pictures and to prevent their deterioration. It was usually done in the most severely affected lobe radiologically.

## Results

Of the total of 900 cases that were referred with recurrent chest infection to pulmonary clinic during the period January 1993 to August 2005, 200 patients were diagnosed to have cystic fibrosis (CF). Of the remaining 700, 151 cases were diagnosed as Non-CF bronchiectasis based on high resolution CT of the chest on 145 (96%) of the patients and chest X-ray in 6 (4%) of the patients due to severe bilateral cystic dilatation of bronchi. Seventy-five (49.7%) were male, 76 (50.3%) were female. One hundred forty-eight (98%) are alive and 3 (2%) died. One hundred forty-four (95%) were Saudi and 7 (5%) were non-Saudi. One hundred forty (93%) were full term. Twenty-two (14.6%) were from the eastern region, 26 (17.2%) from the central region, 39 (25.8%) from the western region, 33 (21.9%) from the southern region and 4 (2.6%) from neighboring countries. Ninety-eight (65%) of the families were consanguineous, while 18 patients (12%) had 1–2 sibling(s) with bronchiectasis and 5 patients had 3-4 siblings with similar disease. Age when symptoms began to develop was 2.3 ± 2.2 years. Age at referral to our center was 6.3 ± 4 years. Age at bronchiectasis diagnosis was 7.3 ± 4.1 years. There was a period of 5 ± 3.2 years between the start of symptoms and the diagnosis of bronchiectasis. Period of follow-up was 5.5 ± 3.9 years.

### Clinical presentations

More than two-thirds of the patients presented with cough, tachypnea, wheezing, sputum production and failure to thrive. Clubbing was found in 50 (33%) of the patients. Cyanosis and oxygen requirement were reported in 35 (23%) of the patients. Hemoptysis was reported only in 7 (5%) of the cases.

### Associated diseases

**Figure 1:**
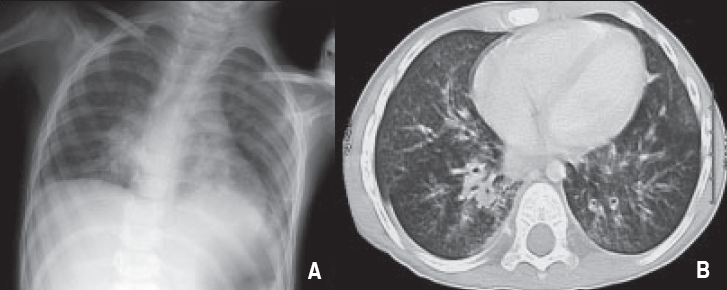
A patient with seizure, brain atrophy, swallowing incoordination, Gastroesophgeal reflux and history of recurrent aspiration pneumonia/ Gastrostomy tube feeding. A. Chest X-ray: Diffuse airspace disease with infiltrates seen mainly in Peri hilar and basal parts. B. CT chest: Extensive bronchial wall thickening with peribronchial inflammatory changes throughout both lungs. peribronchial infiltrate in RLL and LLL with dilated bronchi in both lobes.

**Figure 2:**
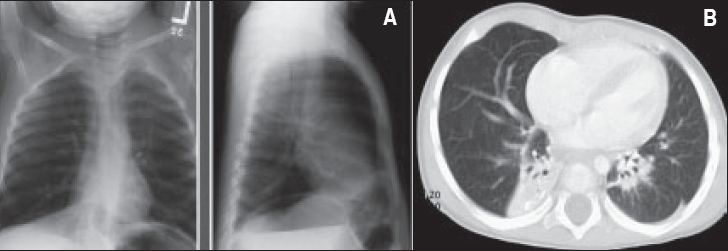
A patient with Spastic diplegia, brain atrophy, respiratory syncytial virus infection, gastroesophageal reflux and bilateral bronchiectasis. A- Chest X-ray: Increase density in lower lobes bilaterally. B. CT chest: Dilatation of bronchi of both lower lobes with collapse/ consolidation of affected lobes.

**Figure 3:**
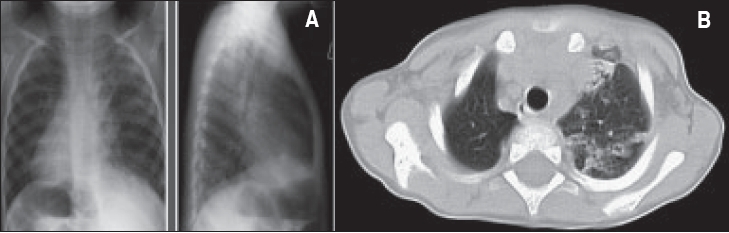
A patient with Karatgener syndrome and bilateral bronchiectasis 3A- Chest X-ray: Dextro cardia, right sided aortic arch. Diffuse air space disease through both lungs. Areas of hyper inflated lung between involved parts, which is typical of chronic lung disease. B. CT chest: Dextrocardia with inversion of abdominal viscera (situs inversus). Extensive chronic lung disease predominantly in the left lung with volume loss in the LUL, with patchy areas of atelectasis and diffuse bronchial dilatation.

Ninety-one (60%) had associated diseases [Table 1]. Pulmonary diseases were found in 48 (32%), immunodeficiency in 27 (18%), CNS anomalies in 10 (7%) [Figures 1 and 2], cardiac in 10 (7%) [Figure 3], skeletal anomalies in 10 (7%) and asthma in 103 (68%) of the patients [Table 1]. More than two-thirds of the patients had two or more associated diseases. In 60% of the patients (40%), no associated disease could be found.

**Table 1:** Bronchiectasis and disease association (total 151 patients)

Disease association	Number (%)
**Pulmonary**	
Asthma	108 (68)
Kartagener	4 (3)
Foreign body aspiration	6 (4)
Immotile cilia syndrome site	17 (11)
Lipid pneumonia	7 (5)
Interstitial pneumonia	2 (1.3)
ABPA	2 (1.3)
Tuberculosis	2 (1.3)
RML syndrome	1 (0.6)
TEF repair	4 (3)
Bronchogenic cyst	7 (5)
Lung collapse	3 (2)
Prematurity	3 (2)
Cardiac diseases:	
Dextrocardia	4 (3)
Congestive heart failure	1 (0.6)
Ventricular septal defect	2 (1.3)
Atrial septal defect	1 (0.6)
Pulmonary hypertension	1 (0.6)
Mitrale valve prolapse	1 (0.6)
Skeletal:	
Pectus excavatum	2 (1.3)
Scoliosis	4 (3)
Absent ribs	3 (2)
Marfan's syndrome	1 (0.6)
**Immunodefficiency**	
Hypogammaglobulinemia	3 (2)
SCIDS	3 (2)
Human immunodeficiency virus	1 (0.6)
Hyper IgE	1 (0.6)
IgG subclass defficiency	6 (4)
Hyper IgM	2 (1.3)
Whiscott Aldrich syndrome	1 (0.6)
Poor antibodies response	4 (3)
Common variable-	3 (2)
Hypogammaglobulinemia	3 (2)
T-cell deficiency	1 (0.6)
Barre lymphocyte syndrome	
Central nervous system disease:	
Cerebral palsy/ seizure disorder	4 (3)
Apnea	1 (0.6)
Craniosynostosis	1 (0.6)
Cutis laxa/ developmental delay	1 (0.6)
Down syndrome/seizure	2 (1.3)
Fatty acid oxidation defect	1 (0.6)
Other disease associations:	
Neuroblastoma	1 (0.6)
Antithrombin III defficiency	2 (1.3)
Corrosive ingestion	2 (1.3)
Liver cirrhosis	1 (0.6)
Ethmoid mucocele	1 (0.6)
Bulllous skin lesion/septicemia	1 (0.6)

FBA - Foreign body aspiration, ABPA - Allergic bronchopulmonary aspergillosis, TB - Tuberculosis, RML - Right middle lobe, TEF - Tracheoesophageal fistula, SCIDS - Severe combined Immunodefficiency

### Radiological investigations

**Figure 4:**
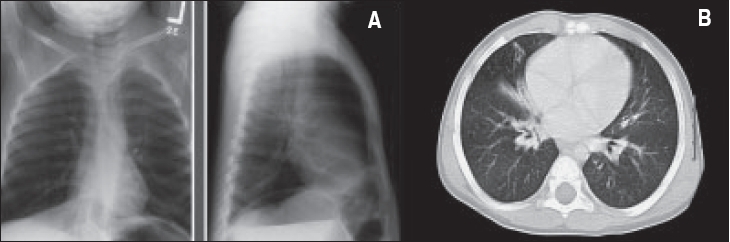
A patient with RML syndrome. A. Chest X-ray: Shows generalized bronchial wall thickening with evidence of peribronchial infiltrate, most marked in the hilar region. Area of atelectasis in the right middle lobe with loss of definition of the right heart border. B. CT chest: subcarinal lymphadenopathy. The RML irregular small bronchi, which are dilated and associated with consolidation.

Consolidation of one or two lobes was found in 137 (91%) of the patients, compensatory hyperinflation in 103 (68%), interstitial pattern in 49 (33%), atelectasis in 117 (78%), peribronchial wall thickening in 115 (76%) and lymph node enlargement of the paratracheal region in 33 (22%) of the patients. Left lower lobe (LLL) was commonly involved in 114 (76%), right middle lobe (RML) in 82 (54%) [Figure 4] and right lower lobe (RLL) in 76 (50%) of the patients; lingula in 73 (48%), right upper lobe (RUL) in 39 (26%) and left upper lobe (LUL) in 27 (18%) of the patients. Unilateral lobar involvement was found in 39 (29%) of the patients and bilateral lobar involvement in 112 (71%). More than two-thirds of the patients had more than two lobes involved with bronchiectasis. In 48 patients (32%) two lobes were involved with bronchiectasis, three lobes were involved in 38 (25%) patients, four lobes in 24 (16%), five lobes in 7 (4.5%) and six lobes in 8 (5.5%) patients. Bronchoscopy was done in 20 of the 117 patients who had persistent atelectasis of the affected lobes; it showed no evidence of foreign body aspiration. The remaining 97 patients had partial improvement of the atelectatic lobes. A total of 102 patients had sinus X-ray, and 18 (12%) had CT sinuses. Sixty-eight (67%) of the 102 patients ‘who had sinus radiological investigations’ were found to have sinusitis. Gastroesophageal reflux (GER) was diagnosed in 49 (32%) of the patients: 33 patients by barium swallow alone, 10 patients by milk scan alone and 6 patients by both radiological procedures. Twenty-two (45%) of the 49 patients with GER required Nissen fundo-plication.

### Type of organisms

**Figure 5:**
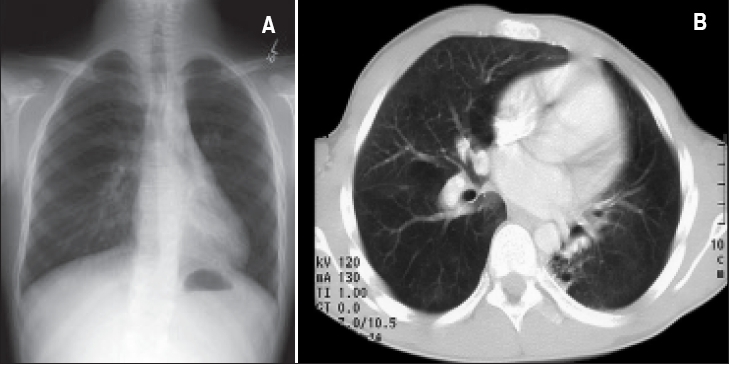
A patient with TB infection proved by lung biopsy and sputum culture and LLL bronchiectasis. A. Chest C-ray: Left lower lobe showing evidence of airway disease with bronchiectasis, bronchial wall thickening and minor peribronchial infiltrate. B. CT chest: Bronchiectatic changes in the left lower lobe, superior and anterior segments. Some areas of fibrosis with contraction of the left lung.

Respiratory cultures were done in 105 (70%) of the patients by sputum cultures as the patients were able to do it or by nasopharyngeal aspirates for patients less than 4 years of age. *Mycobacterium tuberculosis* was cultured in 1 patient [Figure 5]. Hemophilus influenza (H-flue) was cultured in 56 (37%), *Streptococcus pneumoniae* in 25 (17%), *Pseudomonas aeruginosa* in 24 (16%), *Branhamella cattarrhales* in 13 (9%), *Staphylococcus aureus* (Staph.) in 11 (7%) and methicillin-resistant *Staphylococcus aureus* (MRSA) in 3 (2%) of the patients. *Candida albicans* was cultured in 2 (1%) of the patients. Almost 50% of the patients had more than one organism simultaneously. Viral cultures were done in 33 (22%) of the patients: Respiratory syncytial virus in 3 (9%) patients [Figure 2] and enterovirus in 1 (3%) patient.

### PFT

Seventy-seven (49%) of the patients were able to do pulmonary function test (PFT). Sixty-eight (88%) of them had abnormal PFT changes. Seventeen (22%) had obstructive lung changes, 14 (18%) had restrictive lung changes and 37 (48%) had combined obstructive and restrictive lung changes [Table 2]. Sixteen (21%) had mild air follow limitation, 30 (39%) had moderate air follow limitation and 22 (28.5%) had severe air follow limitation. Male patients showed more severe PFT changes at presentation compared to females (*P*< 0.05) [Table 3].

**Table 2:** Pulmonary function test values at presentation (total 77 patients)*

Variable	Mean (SD)	Median	Rang
FVC	66.7 (18.5)	67	28-114
FEV1	64.8 (20.3)	66	33-124
FEV1 / FVC	98 (15.6)	100	41–121
MMEF 25-75%	53 (27.1)	52	9-118
PEF	66.5 (20.3)	67	13-112
% change VENT in FEV1	15.6 (22.9)	9.5	(−31)-98
% change VENT in MMEF 25-75%	24.2 (23.9)	24	(−10)- 65
FRC	106.3 (20.7)	107	64-156
RV	151.5 (40.1)	144.5	80-237
TLC	84.5 (11.6)	84.5	53-109
RV/TLC ratio	46.4 (12.1)	44.5	27-77
RV/TLC (%)	179.9 (44.9)	170.5	101-288

* All values are in percent predicted for age height. FVC - Forced expiratory volume in one second, MMEF - maximum mid-expiratory flow, PEF - Peak expiratory flow. % Ventolin - percentage of change in FEV1 values after administration of Ventolin, FRC - Functional residual capacity, RV - Residual volume, TLC - Total lung capactiy, RV/TLC - The ratio of RV:TLC in percentage and actual values

**Table 3:** Correlation of pulmonary function test severity to different factors (total 77 patients)

Factors	Mild (%)	Moderate/changes severe (%)	Total (%)	*P* value
Male	8 (21)	30 (79)	38 (100)	0.05
Female	16 (41)	23 (59)	39 (100)	
Total	24 (31)	53 (69)	77 (100)	
Sinusitis	14 (32)	30 (68)	44 (100)	0.8
GER	9 (38)	15 (62)	24 (100)	0.4
Ventilation	3 (43)	4 (57)	7 (100)	0.4
Disease association	9 (24)	28 (76)	37 (100)	0.2
Asthma	18 (32)	39 (68)	57 (100)	0.8
Required lobectomy	3 (18)	14 (82)	17 (100)	0.1
O_2_ requirement	1 (14)	6 (86)	7 (100)	0.3
Consanguinity	12 (25)	36 (75)	48 (100)	0.1

GER - Gastroesophageal ruflus, Mild PFT changes - FEV1 65-75% of predicted values for age and height, Moderate/severe PFT changes - FEV1 35-% of predicted values for age and height

### Prognosis and follow-up

Disease progression developed in 72 (48%) of the patients, and it was related to development of symptoms before 5 years of age, persistent atelectasis of the affected lobes and involvement of RLL with bronchiectasis (*P*<0.05). Only 1 case (0.4%) post foreign body aspiration showed marked regression of X-ray changes. The other 78 cases (51.6%) showed clinical improvement but no radiological regression of disease.

Progression of disease was found in 72 (48%) patients. Associated diseases were found in 71 patients. Asthma was found in 54 patients, ciliary dyskinesia in 9 patients, central nervous system anomalies in 2 patients, immunodeficiency in 21 patients, skeletal anomalies in 5 patients, lipid pneumonia in 7 patients, tracheoesophageal fistula in 2 patients, tuberculosis in 2 patients and cardiac anomalies in 4 patients.

Unilateral lobectomy was done in 21 (14%) of the patients, whereas bilateral lobectomies were done in 3 (2%) of them. Follow-up of patients who had lobectomy showed that in 16 of the 21 patients, clinical, radiological and PFT status had improved, while 5 of them had deteriorated due to other associated diseases. Recurrent otitis media was reported in 12 (8%) of the patients. All 3 patients who died had developed acute lung infection in a local hospital that required ventilation and progressed to respiratory failure and death. One of them, who died at 16 years of age, had repair of esophageal atresia and tracheoesophageal fistula with esophageal-colonic anastomosis and recurrent aspiration that required right pneumonectomy. The second patient had type IgM syndrome with lympho-proliferative disorder and CMV infection. The third patient had lipid pneumonia that was complicated with bilateral necrotizing pneumonia, bilateral pneumothoraces, chronic ventilation that required tracheostomy, acute hepatitis, staphylococcal septicemia and recurrent pleural effusion and died at 4 years of age.

## Discussion

Published reports from some developing countries suggest that childhood bronchiectasis may not be disappearing and that it represents a more common problem than in developed countries.[13] Karakoc from Turkey described 23 children with bronchiectasis and found that factors other than infections have contributed to the development of bronchiectasis, such as immunodeficiency, primary ciliary dyskinesia and asthma[2] [Table 4]. A report by Dawson from the Abu Dhabi region of United Arab Emirates described 32 children with bronchiectasis from a population of 300,000.[14] He found that congenital anomalies of the respiratory system, prematurity, immunodeficiency were some of the factors that contributed to the cause of the disease in addition to viral or bacterial infections[14] [Table 4].

**Table 4:** Comparisons of disease associations with other developing countries

Associated disease	Banjar 2005 KSA Total (151 patients) (%)	Karakoc 2001 Turkey Total (23)[2](%)	Dawson 1996 Abu-Dhabi/UAE Total (32)[9](%
Cardiac	12 (8)		
Pulmonary	48 (32)	8 (34)	6 (19)
Kartagener syndrome	5 (3)	3 (13)	
Infections	77 (51)	8 (34)	7 (22)
Central nervous system	18 (12)		
Foreign body aspiration	6 (4)		
Immunodefficiency	27 (18)		3 (9)
Skeletal	10 (7)		
Asthma	103 (68)	4 (17)	
Other	78 (52)		

KSA - Kingdom of Saudi Arabia, UAE - United Arab Emirates

In our report, incidence of bronchiectasis was found to be 1 in 4 cases that presented with recurrent chest infection at our center, which makes it a common problem in this part of the world. In case of bacterial infections, common respiratory organisms such as Staph aureus, H-flue, pneumococcus and pseudomonas were found to be the common bacteria cultured from 51% of the patients. The southwestern region accounted for 50% of the reported cases. Environmental factors such as humidity and crowding during pilgrimage time may have contributed to such increase in its incidence in the western region. Other factors that may have contributed to its high incidence in the southern region could be delayed presentation and treatment as a result of poor living conditions and living in remote areas away from medical facilities.[13,15] Referral bias cannot be excluded as a contributing factor for the high incidence in such areas. Recurrent aspiration pneumonia due to CNS anomalies or seizure is described for the first time in the literature and might be related to recurrent aspiration of secretions due to swallowing incoordination and/or GER. Our report agrees with the other report of early start of symptoms - before 5 years of age in 83% of our population.[1,2,13,15] With cumulative effect for 5 years post-infection before the diagnosis of bronchiectasis is established,[1,13,15] it can be concluded that bronchiectasis may start as an upper or lower respiratory tract infection as flue-like illness triggered by a virus such as adenovirus or respiratory syncytial virus, followed by a secondary infection with bacteria such as mycoplasma or Hemophilus-influenza or others that may cause accumulative lung injury, which, if not treated properly, may lead to permanent lung damage with decreased mucous clearance, obstruction to bronchi and later, bronchiectasis. This process of cumulative lung damage may take 5-10 years to be established.[1,13,15] In one study among 46 Alaskan children reported with bronchiectasis, each child experienced an average of nine lower respiratory tract illnesses before the diagnosis of bronchiectasis was made,[15] and their chest radiographs suggest that lobes that were abnormal at 2 years of age were more likely to become bronchiectatic than a lobe involved during any single infection. Presumably, multiple bacterial and/or viral infections lead to cumulative airway injury, narrowing and poor mucous clearance, setting the stage for evolution of bronchiectasis several years later.[1,13,15] Most of the patients had bilateral lobar involvement and severe PFT changes at presentation. Fifty percent of our patients had radiological and clinical progression in spite of medical treatment of antibiotic prophylaxis, which may suggest the adoption of surgical intervention in patients with progressive disease as lobectomy had been done in only 16% of our population compared to 60-70% in other reports.[2,14–18] Asthma was the common association in 68% of the patients, which is in accordance with other reports,[19–21] and treatment with inhaled steroid and B2 agonist may need to be considered in some patients. Immunodeficiency was found to be the second most common disease association after pulmonary disease in our study [Table 1]. Sinusitis was also a common presentation in our patients (68%), and such patients may need to be treated for a longer period of time, as suggested by other reports for 4-6 weeks.[3] Persistent atelectasis of the affected lobe has been contributing to the development of bronchiectasis in our population, which may warrant encouragement of chest physiotherapy and postural drainage in patients with such a problem. Atelectasis is commonly found in many patients with pneumonia, aspiration or asthma, and repeat chest X-ray should be done after clinical improvement to ensure the re-expansion of the atelectatic part of the lung. Gastroesophageal reflux and recurrent aspiration was found in 32% of our patients and may have contributed to the development of bronchiectasis or complicated its progression.[22] Lobectomy was done in only 16% of our patients compared to 60-70% in other reports, which is considered to be a small proportion in view of the excellent improvement of clinical picture in three-fourths of our patients who had lobectomy (16 out of 21) and may need to be considered early if medical treatment failed to improve clinical or radiological pictures.[16–19] A case control study needs to be done to identify the actual risk factors of developing such disease in our country, and efforts should be made for making early diagnosis, creating awareness of contributing factors and providing early treatment or referral before development of progression.

## References

[1] Callahan CW, Redding G (2002). Bronchiectasis in children: Orphan disease or persistent problem?. Pediatr Pulmonol.

[2] Karakoc GB, Yilmaz M, Altintas DU, Kendiri SG (2001). Bronchiectasis: Still a problem. Pediatr Pulmonol.

[3] Brown MA, Leman RJ, Chernick V, Boat T (1998). Bronchiectasis. Kendig's disorder of the respiratory tract in children.

[4] Biering A (1956). Childhood pneumonia, including pertussis, pneumonia and bronchiectasis: A follow-up study of 151 patients. Acta Pediatr.

[5] Ruberman W, Shaufer I, Bioondo T (1957). Bronchiectasis and acute pneumonia. Am Rev Tuber.

[6] Field CE (1969). Bronchiectasis: 3^rd^ report on a follow-up study of medical and surgical cases from childhood. Arch Dis Child.

[7] Clark NS (1963). Bronchiectasis in childhood. Br Med J.

[8] Dawson KP, Bakalinova D (1997). Childhood chronic lung disease in the United Arab Emirates. Trop Doct.

[9] Banjar H (2005). Bronchiectasis following repair of esophageal atresia and tracheesophgaeal fistula. Saudi Med J.

[10] Annobil SH, Morad NA, Kameswaren M, El Tahir MI, Adzaku F (1996). Bronchiectasis due to lipid aspiration in childhood: Clinical and pathological correlates. Ann Trop Pediatr.

[11] Saquib Malick M, Rauf Khan A, Al-Bassam A (2005). Late presentation of tracheobronchial foreign body aspiration in children. J Trop Pediatr.

[12] Al-Mobaireek AF, Al-Sahrani A, Al-Amri S, Bamgboye E, Ahmed SS (2002). Chronic cough at a non-teaching hospital: Are extrapulmonary causes overlooked?. Respirology.

[13] Barker AF, Bardana EJ (1988). Bronchiectasis: Update of an orphan disease. Am Rev Respir Dis.

[14] Dawson KP, Bakalinova D (1996). Child bronchiectasis in a desert location. Middle East Pediatr.

[15] Singleton R, Morris A, Redding G, Poll J, Holck P, Martinez P (2000). Bronchiectasis in Alaska Native children: Causes and clinical courses. Pediatr Pulmonol.

[16] Dogan R, Alp M, Kaya S, Ayrancioglu K, Tastepe I, Unlu M (1988). Surgical treatment of bronchiectasis: A collective review of 487 cases. Thorac Cardiovasc Surg.

[17] Wilson JF, Decker AM (1982). The surgical management of childhood bronchiectasis. Ann Surg.

[18] Fujimoto T, Hillejan L, Stamatis G (2001). Current strategy for surgical management of bronchiectasis. Ann Thorac Surg.

[19] Ip MS, So SY, Lam WK, Yam L, Liong E (1992). High prevalence of asthma in patients with bronchiectasis in Hong Kong. Eur Respir J.

[20] Bahous J, Cartier A, Pineau L, Bernard C, Ghezzo H, Martin RR (1984). Pulmonary function test and airway responsiveness to methacholine in chronic bronchiectasis of the adults. Bull Eur Physiopath Respir.

[21] Varpela E, Laitinen LA, Keskinen H, Korhola O (1978). Asthma, allergy and bronchial hyper-reactivity to histamine in patients with bronchiectasis. Clin Allergy.

[22] El-Serag HB, Gilger M, Kuebeler M, Rabeneck L (2001). Extraesophageal association of gastroesophageal reflux disease in children without neurologic defect. Gastroenterology.

